# Supplementary material for: Short-term effects of side-alternating Whole-Body Vibration on cognitive function of young adults
Source: PLoS One. 2023 Jan 12;18(1):e0280063. doi: 10.1371/journal.pone.0280063 (PMC9836316; doi:10.1371/journal.pone.0280063)
Supplement: S1 Dataset — (DOCX) [file pone.0280063.s001.docx]

| Sit_Stroop_Vib_1 | Sit_Stroop_Con_1 | Sit_Stroop_Vib_2 | Sit_Stroop_Con_2 | Sit_Stroop_Vib_3 | Sit_Stroop_Con_3 | Stand_Stroop_Vib_1 | Stand_Stroop_Con_1 | Stand_Stroop_Vib_2 | Stand_Stroop_Con_2 | Stand_Stroop_Vib_3 | Stand_Stroop_Con_3 |
| --- | --- | --- | --- | --- | --- | --- | --- | --- | --- | --- | --- |
| 37,05 | 35,57 | 32,5 | 34,92 | 32,6 | 32,32 | 28,07 | 29,01 | 27,37 | 28,13 | 28,07 | 28,33 |
| 38,63 | 36,53 | 39,69 | 33,63 | 36,74 | 36,09 | 42,05 | 41,77 | 40,95 | 39,2 | 47,38 | 46,31 |
| 42,06 | 43,38 | 47,38 | 46,48 | 38,54 | 44,44 | 44,62 | 38,63 | 36,46 | 38,46 | 44,08 | 51,09 |
| 33,16 | 32,25 | 34,48 | 32,49 | 33,71 | 32,11 | 38,97 | 35,59 | 36,9 | 34,94 | 36,44 | 33,88 |
| 35,19 | 36,67 | 36,03 | 33,64 | 32,89 | 32,76 | 31,81 | 30,87 | 28,97 | 29,44 | 31,81 | 31,58 |
| 33,76 | 33,41 | 29,23 | 32,89 | 31,59 | 30,59 | 28,25 | 30,76 | 26,9 | 28,85 | 29,85 | 29,96 |
| 38,16 | 40,6 | 46,19 | 42,9 | 36,78 | 43,56 | 37,81 | 42,98 | 46,9 | 37,87 | 37,41 | 37,88 |
| 30,27 | 34,68 | 32,43 | 35,43 | 32,55 | 32,18 | 30,4 | 31,76 | 30,88 | 34,26 | 33,92 | 34,2 |
| 37,96 | 38,26 | 34,06 | 32,19 | 31,08 | 30,78 | 35,09 | 36,53 | 34,63 | 33,29 | 36,01 | 42,18 |
| 27,76 | 34,06 | 32,31 | 29,55 | 30,51 | 30,12 | 34,65 | 37,35 | 34,27 | 35,72 | 33,97 | 36,71 |
| 39,92 | 37,57 | 39,79 | 34,59 | 38,68 | 39,3 | 43,95 | 43,26 | 42,26 | 43,08 | 41,46 | 42,63 |
| 25,93 | 28,25 | 25,33 | 26,28 | 28,52 | 27,11 | 29,12 | 30,19 | 29,75 | 30,22 | 26,26 | 26,21 |
| 23,77 | 26,22 | 20,08 | 22,62 | 20,62 | 22,59 | 30,45 | 29,69 | 30,36 | 28,34 | 24,98 | 26,91 |
| 27,5 | 31,24 | 26,81 | 27,19 | 26,47 | 26,44 | 22,78 | 26,13 | 25,32 | 25,03 | 23,71 | 22,72 |
| 28,78 | 31,6 | 30,44 | 32,51 | 30,81 | 31,68 | 32,1 | 34,9 | 32,41 | 32,92 | 29,43 | 32,88 |
| 28,76 | 30,62 | 30,24 | 28,22 | 29,09 | 29,93 | 27,2 | 29,13 | 30,22 | 27,38 | 25,78 | 27,27 |
| 24,15 | 26,99 | 28,08 | 27,42 | 27,71 | 26,57 | 39,13 | 29,23 | 29,93 | 26,93 | 29,58 | 26,92 |
| 39,51 | 35,82 | 38,07 | 37,15 | 34,95 | 36,69 | 44,74 | 35,33 | 34,79 | 33,91 | 34,14 | 31,88 |
| 38,67 | 36,6 | 36,8 | 40,32 | 35,83 | 38,36 | 48,53 | 45,35 | 41,83 | 42,21 | 49,06 | 43,57 |
| 33,77 | 34,58 | 30,62 | 31,01 | 30,72 | 35,39 | 28,45 | 27,65 | 29,2 | 30,77 | 25,64 | 25,48 |
| 28,47 | 28,89 | 27,68 | 26,51 | 27,89 | 27,16 | 24,2 | 22,35 | 24,02 | 25,31 | 24,2 | 23,14 |
| 27,03 | 31,35 | 27,56 | 30,77 | 28,41 | 28,39 | 31,58 | 35,66 | 33 | 30,62 | 30,94 | 33,51 |
| 36,32 | 35,44 | 31,4 | 29,75 | 28,42 | 35,06 | 32,46 | 31,05 | 35,85 | 31 | 30,35 | 29,14 |
| 29,99 | 23,67 | 23,3 | 26,25 | 25,48 | 26,35 | 29,45 | 29,41 | 28,48 | 29,08 | 27,95 | 28,03 |
| 42,93 | 45,77 | 48,44 | 52,24 | 48,16 | 51,24 | 41,08 | 49,33 | 37,72 | 44,1 | 39,82 | 40,07 |
| 24,45 | 27,24 | 27,14 | 26,28 | 27,86 | 24,55 | 31,55 | 33,93 | 33,41 | 32,73 | 29,72 | 33,89 |
| 30,59 | 33,2 | 36,08 | 32,98 | 35,06 | 31,97 | 30,38 | 29,18 | 31,14 | 32,64 | 43,32 | 34,98 |
| 35,4 | 37,29 | 36,41 | 33,54 | 31,19 | 35,58 | 28 | 29,12 | 29,57 | 27,97 | 32,55 | 29,65 |
| 30,41 | 30,57 | 31 | 29,47 | 26,94 | 29,45 | 37,29 | 35,31 | 31,08 | 29,77 | 30,61 | 30,34 |
| 31,48 | 31,57 | 30,24 | 34,59 | 31,54 | 34,63 | 32,67 | 32,42 | 28,27 | 31,14 | 30,65 | 32,65 |
| 36,77 | 37,73 | 45,91 | 40,34 | 37,57 | 37,03 | 32,23 | 32,25 | 35,96 | 34,17 | 33,08 | 33,54 |
| 52,69 | 56,73 | 49,62 | 55,82 | 54,2 | 54,57 | 63,18 | 58,2 | 47,51 | 53,36 | 48,31 | 48,76 |
| 44,12 | 49,89 | 43,58 | 40,36 | 45,33 | 36,6 | 37,06 | 38,83 | 33,02 | 36,26 | 36,81 | 38,46 |
| 33,95 | 33,71 | 33,2 | 36,78 | 35,63 | 34,25 | 39,11 | 43,28 | 40,18 | 39,27 | 39,61 | 40,65 |
| 36,55 | 36,28 | 32,21 | 33,21 | 31,84 | 33,23 | 27,91 | 28,88 | 28,2 | 30,01 | 29,01 | 29,23 |
| 26,89 | 27,37 | 28,56 | 28,54 | 26,89 | 27,58 | 30,44 | 28,84 | 31,07 | 28,03 | 29,23 | 30,07 |
| 30,48 | 27,99 | 27,48 | 31,29 | 27,57 | 28,92 | 31,6 | 34,5 | 32,06 | 31 | 34,47 | 30,23 |
| 29,16 | 30,91 | 27,23 | 31,62 | 24,8 | 26,71 | 28,07 | 26,62 | 26,02 | 25,49 | 25,36 | 23,47 |
| 46,96 | 47,49 | 42,55 | 38,21 | 39,49 | 42,54 | 37,01 | 40,31 | 38,9 | 37,82 | 34,69 | 43,15 |
| 35,51 | 35,37 | 34,25 | 32,42 | 31,79 | 35,85 | 31,39 | 32,97 | 32,36 | 28,9 | 31,37 | 30,16 |
| 32,26 | 34,82 | 33,8 | 32,99 | 33,12 | 34,8 | 36,1 | 35,3 | 35,32 | 35,32 | 34,52 | 35,98 |
| 33,39 | 30,7 | 29,84 | 31,95 | 26,07 | 26,04 | 26,07 | 26,04 | 24,84 | 21,95 | 29,87 | 23,59 |
| 30,08 | 37,75 | 29,84 | 32,58 | 29,69 | 31,19 | 41,8 | 36,29 | 35,61 | 34,39 | 39,59 | 39,49 |
| 33,76 | 29,93 | 28 | 32,54 | 29,15 | 29,18 | 29,55 | 26,45 | 28,38 | 29,16 | 26,81 | 28,41 |
| 31,31 | 36,71 | 32,78 | 30,81 | 29,67 | 29,5 | 38,46 | 36,14 | 33,49 | 33,19 | 33,54 | 30,85 |
| 27,86 | 32,74 | 29,92 | 30,65 | 32,29 | 29,99 | 37,64 | 37 | 34,2 | 33,9 | 32,27 | 36,14 |
| 27,89 | 29,59 | 28,3 | 29,74 | 31,43 | 30,18 | 29,55 | 28,99 | 28,76 | 32,05 | 28,29 | 29,69 |
| 30,02 | 32,18 | 30,93 | 34,18 | 29,51 | 30,37 | 37,85 | 36,54 | 35,42 | 34,55 | 36,38 | 34,2 |
| 40,27 | 36,86 | 36,99 | 35,35 | 36,04 | 37,19 | 35,65 | 31,54 | 38,18 | 38,77 | 34,21 | 33,77 |
| 33,83 | 39,19 | 35,45 | 29,3 | 37,81 | 30,05 | 29,28 | 26,97 | 27,87 | 34,68 | 30,31 | 27,15 |
| 35,18 | 30,97 | 32,17 | 30,4 | 31,84 | 34,27 | 34,48 | 37 | 36,31 | 37,51 | 35,52 | 33,37 |
| 42,24 | 39,33 | 38,15 | 38,06 | 37,08 | 41,07 | 39,42 | 38,96 | 37,89 | 39,77 | 40,31 | 38,14 |
| 44,48 | 42,23 | 36,18 | 35,38 | 44,67 | 44,28 | 42,25 | 42,23 | 40,54 | 38,11 | 37,51 | 40,93 |
| 31,51 | 29,78 | 28,57 | 31,13 | 24,73 | 25,85 | 26,4 | 24,26 | 35,67 | 27,25 | 27,03 | 25,25 |
| 42,43 | 47,02 | 45,4 | 45,81 | 47,4 | 44,54 | 51,59 | 46,74 | 58,05 | 53,29 | 51,27 | 55,12 |
| 25,15 | 25,41 | 22,28 | 24,12 | 27,62 | 22,8 | 28,34 | 31,75 | 26,87 | 29,62 | 25,92 | 26,28 |
| 42,78 | 38,41 | 41,19 | 39,45 | 38,01 | 41,55 | 40,11 | 48,61 | 53,36 | 44,32 | 48,79 | 40,38 |
| 38,76 | 36,34 | 41,6 | 36,17 | 30,91 | 31,42 | 48,42 | 44,06 | 44,06 | 43,62 | 42,16 | 41,42 |
| 33,41 | 36,81 | 29,44 | 28,43 | 27,13 | 30,65 | 39,62 | 36,23 | 35,26 | 36,39 | 34,65 | 30,15 |
| 28,12 | 31,13 | 31,66 | 30,29 | 28,25 | 30,81 | 30,79 | 33,16 | 31,86 | 31,31 | 24,73 | 25,85 |
