# Supplementary material for: Short-term effects of side-alternating Whole-Body Vibration on cognitive function of young adults
Source: PLoS One. 2023 Jan 12;18(1):e0280063. doi: 10.1371/journal.pone.0280063 (PMC9836316; doi:10.1371/journal.pone.0280063)
Supplement: Attachment — Submitted filename: Response to Reviewers.docx [file pone.0280063.s003.docx]

**Rebuttal letter PONE-D-22-16452**

Dear academic editor and reviewers,

We want to thank the academic editor and reviewers for their effort to review our manuscript “Short term effects of side-alternating Whole-Body Vibration on cognitive function of healthy young adults”. We carefully revised our manuscript with and without track changes. Please find below our response.

**General remarks**

| Remark | Response |
| --- | --- |
| 1. Please ensure that your manuscript meets PLOS ONE's style requirements, including those for file naming. | We carefully checked the document including the references and renamed the files. We feel that the manuscript now meets the requirements. |
| 2. Please provide additional details regarding participant consent. In the ethics statement in the Methods and online submission information, please ensure that you have specified what type you obtained (for instance, written or verbal, and if verbal, how it was documented and witnessed). If your study included minors, state whether you obtained consent from parents or guardians. If the need for consent was waived by the ethics committee, please include this information. | We added the fact that we obtained *written* informed content (**line 148**) We did not include minors. |
| Please review your reference list to ensure that it is complete and correct. If you have cited papers that have been retracted, please include the rationale for doing so in the manuscript text, or remove these references and replace them with relevant current references. Any changes to the reference list should be mentioned in the rebuttal letter that accompanies your revised manuscript. If you need to cite a retracted article, indicate the article’s retracted status in the References list and also include a citation and full reference for the retraction notice. | Based on the comments of reviewer 1, we added several references (see below). Thereafter, we checked and corrected our reference list again. We did not cite papers that have been retracted. |
| While revising your submission, please upload your figure files to the Preflight Analysis and Conversion Engine (PACE) digital diagnostic tool, https://pacev2.apexcovantage.com/. PACE helps ensure that figures meet PLOS requirements. To use PACE, you must first register as a user. Registration is free. Then, login and navigate to the UPLOAD tab, where you will find detailed instructions on how to use the tool. If you encounter any issues or have any questions when using PACE, please email PLOS at figures@plos.org. Please note that Supporting Information files do not need this step. | We already used PACE for our first submission. |

**Reviewer 1**

We thank reviewer 1 for his kind words and comments and suggestions to revise the document.

| Comments Reviewer 1  (Line numbers refer to original manuscript) | Response  (Line numbers refer to revised manuscript showing all mark-up) |
| --- | --- |
| **Section: Abstract** | |
| Comment reviewer #1  **Line 26:** Use only WBV | Thank you, we changed Whole-Body Vibration (WBV) into WBV (**line 28**). |
| Comment reviewer #1 **Line 33:** Add p-value | We added the p-value (**line 35)**. |
| **Section: Introduction** | |
| Comment reviewer #1 **Line 49:** I suggest adding references at the end of this paragraph. Some suggestions are:  - Chen T, Liu WB, Ren X, Li YF, Li W, Hang CH, Wang YH. Whole Body Vibration Attenuates Brain Damage and Neuroinflammation Following Experimental Traumatic  Brain Injury. Front Cell Dev Biol. 2022 Apr 7;10:847859. doi:  10.3389/fcell.2022.847859.  -Sá-Caputo DC, Coelho-Oliveira AC, Pessanha-Freitas J, Paineiras-Domingos LL, Lacerda ACR, Mendonça VA, Sonza A, Taiar R, Sartorio A, Seixas A, Bernardo-Filho M. Whole-Body Vibration Exercise: A Possible Intervention in the Management of Post COVID-19 Complications? Applied Sciences. 2021; 11(12):5733  - Nowak-Lis A, Nowak Z, Gabrys T, Szmatlan-Gabrys U, Batalik L, Knappova V. The  Use of Vibration Training in Men after Myocardial Infarction. Int J Environ Res  Public Health. 2022 Mar 11;19(6):3326. doi: 10.3390/ijerph19063326.  - Li KY, Cho YJ, Chen RS. The Effect of Whole-Body Vibration on Proprioception  and Motor Function for Individuals with Moderate Parkinson Disease: A Single-  Blind Randomized Controlled Trial. Occup Ther Int. 2021 Dec 17;2021:9441366.  doi: 10.1155/2021/9441366. PMID: 34992511; PMCID: PMC8709745. | Thank you for your suggestions. We added the references in the correct reference style (**line 49)**. |
| Comment reviewer #1 **Line 57:** I suggest adding the recent reference: Santos LMM, Oliveira ACC, Fonseca SF, Silva AF, Santos JNV, Souza ALC, Santos JM, Ribeiro VGC, Arrieiro AN, Prates ACN, Soares LA, Figueiredo PHS, Martins F, Lima VP, Fernandes JSC, Bernardo-Filho M, Taiar R, Borges DT, Sartorio A, Costa HS, Leite HR, Mendonça VA, Lacerda ACR. Whole-Body Vibration Exercise in Different Postures on Handgrip Strength in Healthy Women: A Cross-Over Study. Front Physiol. 2021 Jan 12;11:469499. doi: 10.3389/fphys.2020.469499 | Thank you for your suggestions. We added the references in the correct reference style (l**ine 57)**. |
| Comment reviewer #1 **Line 57:**  I suggest adding the reference:  Paineiras-Domingos LL, da Cunha Sá-Caputo D, Reis AS, Francisca Santos A,  Sousa-Gonçalves CR, Dos Anjos EM, Dos Santos Pereira MJ, Sartorio A, Bernardo-  Filho M. Assessment Through the Short Physical Performance Battery of the  Functionality in Individuals With Metabolic Syndrome Exposed to Whole-Body  Vibration Exercises. Dose Response. 2018 Sep 4;16(3):1559325818794530. doi:  10.1177/1559325818794530 | Thank you for your suggestions. We added the reference in the correct reference style (**line 57)**. |
| **Line 58:** I suggest adding the references:  - ElDeeb AM, Abdel-Aziem AA. Effect of Whole-Body Vibration Exercise on Power  Profile and Bone Mineral Density in Postmenopausal Women With Osteoporosis: A  Randomized Controlled Trial. J Manipulative Physiol Ther. 2020  May;43(4):384-393. doi: 10.1016/j.jmpt.2019.12.003  -Dionello CF, Sá-Caputo D, Pereira HV, Sousa-Gonçalves CR, Maiworm AI, Morel DS, Moreira-Marconi E, Paineiras-Domingos LL, Bemben D, Bernardo-Filho M. Effects of whole body vibration exercises on bone mineral density of women with postmenopausal osteoporosis without medications: novel findings and literature review. J Musculoskelet Neuronal Interact. 2016 Sep 7;16(3):193-203. PMID: 27609034 | Thank you for your suggestions. We added the references in the correct reference style (**line 58)**. |
| **Line 60:** I suggest finishing this sentence with the references [12-14 and to add the reference:  Cardoso ALBD, Sá-Caputo DC, Asad NR, van Heuvelen MJ, van der Zee EA, Ribeiro-Carvalho A, Bernardo-Filho M. Beneficial effects of whole-body vibration exercise for brain disorders in experimental studies with animal models: a systematic review. Behav Brain Res. 2022 Aug 5;431:113933. doi: 10.1016/j.bbr.2022.113933 | Thank you for your comment. We finished the sentence with the references suggested and we have added the references you have suggested in the correct reference style (**line 62)**. |
| **Line 69:** To define fNIRS in the first time in the manuscript. | We defined fNIRS as Functional near-infrared spectroscopy (**line 70)**. |
| **Line 77:** To define in the first time in the manuscript. | We defined glut 1 as Glucose transporter 1 (**line 79)**. |
| **Line 89:** To define ADHD in the first time in the manuscript. | We defined ADHD as Attention Deficit Hyperactivity Disorder (**line 92).** |
| **Line 112:** To define EEG in the first time in the manuscript | We defined EEG as Electroencephalography and included that the study measured brain activation for clarification (**line 116)**. |
| **Line 114:** To define MCI in the first time in the manuscript | We deleted MCI and rephrased the sentence to make it more readable (**line 118).** |
| **Section: Methods** | |
| **Line 138:** To define CONSORT in the first time in the manuscript | Thank you for your comment. We defined CONSORT as Consolidated Standards of Reporting Trials with additional information. We rephrased the sentences for clarity (**line 132)**. |
| **Line 153:** I suggest using...intervention or procedure | We changed ‘experiment’ into ‘ procedure’ (**line 150)**. |
| **Line 162:** To clarify | We clarified this sentence by explaining that participants did not receive any financial or other compensations for their contribution (**line 156)**. |
| **Line 180 table 1:** I suggest using body mass instead of weight | We changed body weight into body mass as suggested (**line 167)**. |
| **Line 256:** h | We added ‘h’ after the numbers so that it is clear it is about the hours (**line 220)**. |
| **Line 285:** I suggest adding that the participants were wearing socks | Thank you for your comment. In the section ‘general protocol’ we added that the participants had to take off their shoes wearing socks only. We repeated this in the section you mentioned to clarify it (**line 242)**. |
| **Line 339:** Use all the "p" related to statistical analysis in italic format, here and throughout the manuscript | We now report the p-values related to statistical analyses in italic (**line 275)**. |
| **Section: Discussion/Conclusion** | |
| **Line 503:** To delete | We deleted the abbreviation ‘CBT’ (**line 411).** |
| **Line 508:** The current study... | We changed ‘this study’ into ‘the current study’ (**line 416).** |
| **Line 563:** To improve the presentation of this reference carefully | Thank you for your comment. We corrected the reference and we checked all the references again to make sure the references meet the PLOS ONE referencing style (**line 496)**. |
| **Section References** | |
| **Line 592:** Is it necessary the doi in this citation? It is not present in the other citations. | We removed the DOI here (**line 521)**. |
| **Line 648:** To add the title of the jounal | We improved the reference and we checked all the references again to make sure the references meet the PLOS ONE referencing style (**line 568)**. |

**Reviewer 2**

We thank reviewer 2 for his/her kind and supportive words.
